# Programmable Automated System for Songbird Ecobehavioral Research (PASSER): Using flexible computer‐integrated feeders to conduct high resolution studies of environment–behavior dynamics in songbirds

**DOI:** 10.1002/ece3.4638

**Published:** 2018-12-01

**Authors:** Conner Philson, Andrew Ray, Sarah Foltz, Jason Davis

**Affiliations:** ^1^ Department of Biology Radford University Radford Virginia; ^2^ Department of Information Technology Radford University Radford Virginia

**Keywords:** automated data collection, behavior, behavior dynamics, bird feeder, computerized feeder, environment, ethology, feeding patterns, microhabitat, songbird, spatiotemporal variation

## Abstract

Field studies seeking to identify interactions between the environment and behaviors of wild songbirds are often restricted by time, labor, and accessibility of the site; hampering the collection of long‐term, high‐resolution data. Here, we describe the development, utilization, and initial results of a long‐term field study of wild songbird feeding patterns using data collected through an inexpensive microcomputer‐controlled automated feeder. Our studies indicate the “smart feeder” is capable of reliable and accurate data collection on feeding and behavioral metrics over long durations with relation to a wide range of environmental conditions. This enables detailed analysis of songbird's environment–behavior interactions. Our results have identified trends in environment–behavior interactions, microhabitat variations, species‐specific feeding profiles, and differences in the frequency and involvement of displacement events. Computerized feeders enabled us to address environment–behavior interactions, resulting in more detailed data than traditional observational methods. This reinforces conclusions from previous work regarding the potential for automated data collection to be adapted for a wide variety of research studies across the field of ethology.

## 1 INTRODUCTION

Ethological and ecobehavioral field studies are often hampered by the scale and difficulty of data collection. Long periods of time and complex data acquisition procedures are required to compose an accurate picture of environmental‐behavioral interactions at a single site. Expanding this to include concurrent data from multiple sites that may have varying habitats spread across multiple locales exacerbates existing data collection issues and limits the number of populations that can be studied (Bonier et al., 2007).

Identifying how the environment can influence animal behavior becomes even more difficult when the conditions or events being studied are uncommon or unpredictable. For instance, many animals may alter behavioral patterns dramatically in the face of unusual or extreme weather patterns (Bateman et al., 2015; Romero, Reed, & Wingfield, 2000), but observation and data collection during such events are frequently hampered by the same factors. It can be difficult to predict an incoming blizzard or tropical storm, and the harsh conditions of these events can also limit access to sites, impair visibility, and create risks to researcher safety.

Similarly, many research questions may require multiple observations of behavioral events that occur infrequently or unpredictably, such as the initial arrival of individuals at a breeding site, aggressive interactions between heterospecific competitors, or mate choice interactions. In some situations, these events can be studied only by spending large amounts of time onsite waiting for the event to happen (Bennett, 1990; Walther, Chen, Lin & Sun, 2017). Unfortunately, many researchers do not have the time or resources to devote to these sorts of long‐term observations.

In addition, the behaviors researchers seek to study may be fundamentally altered by the act of observation (Burley, Krantzberg, & Radman, 1982; Metz & Weatherhead, 1991). The presence of the researcher, even when minimized through distance, camouflage or cover, creates a potential confound that can alter both the incidence and salient features of behavior (Farmer, Leonard, Mills Flemming, & Anderson, 2014; Gibson, Blomberg, Atamian, & Sedinger, 2015; Jiguet, 2009). Similarly, it is difficult if not impossible to conduct certain types of experimental protocols while avoiding interactions between the researcher and the research subject. For instance, a hypothetical avian study involving playback of predatory vocalizations during chick feeding events would require close observation of parental behavior and a dynamic interaction with a playback apparatus; the researcher would have to watch the nest for the return of the parents and play the predatory vocalization at exactly the right moment. This hypothetical study requires the researcher to be present in relatively close proximity to the nest, creating a potential confound. As a result, this research question becomes not “how do parents react to predatory vocalizations during chick feeding” but rather “how do parents react to predatory vocalizations during chick feeding, when a potential threat (the researcher) is near the nest.”.

Other approaches such as large teams of researchers and/or citizen science can ameliorate some of these difficulties, however come with complications of their own (Dickinson, Zuckerberg, & Bonter, 2010). Citizen Science initiatives may be particularly hampered in that they require buy‐in from an often uninterested public and possess relatively high turnover rates (Bonter & Cooper, 2012). Data collected through citizen science are also vulnerable to substantial accuracy variation as well as uneven spatial and temporal resolutions due to inconsistent distribution and involvement of participants (Farmer et al., 2014).

A technologically driven approach to data collection can provide an alternative, or supplementary, method of addressing the practical problems limiting ecobehavioral research. A number of researchers have integrated computers into their data collection devices, making them capable of reliable, round‐the‐clock, remote ecobehavioral data collection (Lendvai et al., 2015; Small, Bridge, & Schoech, 2011; Venier, Holmes, Holborn, Mcilwrick, & Brown, 2012). Aplin, Farine, Morand‐Ferron, and Sheldon (2012) and Firth and Sheldon (2015) used automated feeding units equipped with RFID (radio‐frequency identification) tags to track individual movements and construct social networks of great tits (*Parus major*), blue tits (*Cyanistes caeruleus*), and other species. Though these feeders were only able to respond to tagged animals, and did not record environmental metrics of the feeding, they did demonstrate that computerized feeders can successfully enable novel types of field data collection.

While many early attempts at computerized field monitoring devices have been generally limited by the nature of the data they collect, their manufacturing expense, and their propensity to errors and critical failures, recent technological developments in embedded computing, 3D printing, and fabrication have made customized, flexible behavioral data collection devices a more reasonable prospect. Solving or minimizing the difficulties inherent in collecting continuous, accurate and fine‐scale data over multiple disparate areas is an increasingly achievable goal that allows researchers to test hypotheses previously left untested due to lack of necessary resources and abilities.

In order to partially address these needs within the field of avian behavioral ecology, we developed the Programmable Automated System for Songbird Ecobehavioral Research (PASSER). The goal of PASSER was to produce a highly reliable, low cost, mobile, and adaptable system that could provide round‐the‐clock monitoring of long‐term species level avian feeding behavior, as well as a variety of potentially salient environmental variables. Here, we describe the methodology and mechanisms of the PASSER “smart feeder” systems we produced, and provide an overview of their data collection capabilities. Specifically, we test the hypotheses that the (a) feeding behaviors of species (both inter‐ and intraspecifically) will vary within sites as well as (b) between sites. We also test the hypothesis that (c) involvement in displacement events will vary across species. Lastly, we compare data collected by our computerized feeders against that of human collected data via field observations to test the hypothesis that (d) our computerized feeders are capable of collecting similar data with greater accuracy than traditional observational methods.

## 2 METHODS

### 2.1 Design

The feeders are rectangular boxes measuring 20.2 × 20.2 × 35.6 cm, made of 0.635 cm thick acrylic with integrated 3D‐printed plastic components. These printed portions provide support to the feeder and house batteries, computer components, sensors, and a motor. We used AutoCAD (Autodesk Inc.) to design the main structure, and TinkerCAD (Autodesk Inc.) for visual modeling and creation of the 3D‐printed parts. The acrylic for the main feeder structure was cut with a ShopBot Computer Numerical Control machine (ShopBot, Durham, USA). 3D components were printed on a Stratus Mojo (Stratasys, Eden Prairie, USA) and a MakerBot Replicator 2 (MakerBot, New York, USA). Feeders were fused with acrylic glue, sealed with silicone, and treated with a hydrophobic spray to keep out water. Each feeder can be powered directly from a wall outlet or by two DC batteries (Voltaic Systems V44 Lithium‐Ion Batteries; Voltaic Systems, New York, USA) that sit atop the feeder. Longevity of the batteries is increased by attaching one or more solar panels (9‐watt Voltaic Solar Cells; Voltaic Systems, New York, USA) positioned atop, or near the feeder. Batteries are currently swapped out every 48–72 hr (depending on the amount of sunlight the solar cells have received) to ensure the feeders remain running.

**Figure 1:**
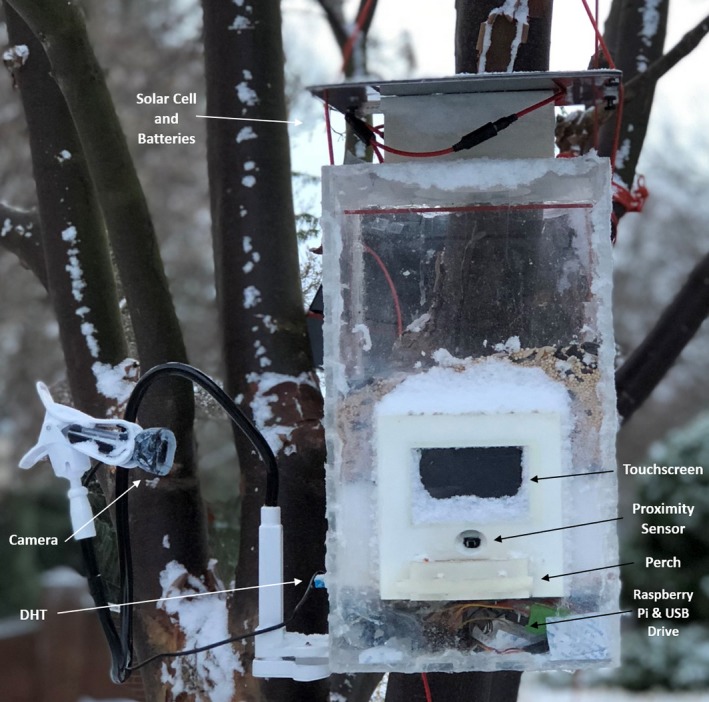
The PASSER smart feeder with key components labeled

Birds interact with two key components while at the feeder, a perch and feed tray. Both of these components are located on the front face of the feeder. In the configuration we currently use, the perch is a rod that is 8.9 cm wide and 0.925 cm in diameter, positioned 4.8 cm from the feed tray. The feeding tray measures 8 × 3.8 × 2.5 cm, providing easy access for small and mid‐sized birds. We use a feed mix comprised of cracked and uncracked sunflower seeds and millet, though this can easily be substituted with other feeds as needed. See Figure 1 for visual.

The feeder is controlled by a Raspberry Pi Zero computer (Raspberry Pi, Cambridge, UK) using the operating system Raspbian (Raspberry Pi, Cambridge, UK) with code written in the BASH scripting language. The Pi is connected to six components: a temperature/humidity sensor (DHT), a servo motor, a proximity sensor, a real‐time clock (RTC), a repositionable armature camera, and a USB drive (Adafruit Industries, New York, USA; Logitech, Romanel‐sur‐Morges, Switzerland). The DHT sensor collects temperature and humidity data from the feeder's immediate microclimate and is located on the side of the feeder. The servo motor, located internally between the feed tray and feed reservoir, spins a wheel, dispensing food from the feed reservoir to the feed tray. The proximity sensor is situated directly above the feed tray, immediately facing the perch, and is triggered upon occlusion of its direct line of sight, within 5 cm. The RTC, which contains a separate internal battery, is used to maintain accurate dates and times. Data collected by the feeder (temperature, humidity, time, date, and images) are saved on the USB drive for easy retrieval and access. The Raspberry Pi, RTC, and USB are located in a sealed, waterproofed compartment below the primary feed reservoir. In addition to these components, an 85 × 50cm touch screen (Adafruit Industries, New York, USA) mounted directly in front of the perch allows for feeder‐bird interactions. The screen is versatile, allowing for predetermined visual media, live feeds from the camera, or interactive stimuli to be displayed in a customizable fashion. General maintenance (replacement of sensors, cleaning the feeder, etc.) was performed as needed, most often at night.

We estimate the price of early feeder prototypes (not including labor or design) cost in the vicinity of $500–$600 USD. Newer designs, based on preexisting templates and proven components, can be produced for an estimated $322 USD, with a majority of costs allocated to batteries, solar cells, 3D printing.

### 2.2 Function

Aggregation of these components allows for the feeder to recognize when a bird arrives, and in turn activates a chain of events that collects both environmental data and images of the feeding bird. The exact chain of events is reconfigurable. In its current iteration, occlusion triggers the servo to dispense food within 2 s and activates the camera, which takes a series of 10 photos across 8.2 s. After the photos, the DHT is activated to record temperature and humidity data, while the built‐in RTC records the date and time of the bird's visit. We have also incorporated a delay between triggering events to minimize the number of times the feeder is triggered by a single feeding event, such that the feeder can only be activated every 11 s.

### 2.3 Field sites and feeder deployment

We placed feeders at three sites (Campus, Residential, and Conservancy) all within a 2.88 km radius in southwest Virginia's New River Valley. Each site was assessed for level of anthropogenic disturbance (Table 1). We quantified disturbance using three metrics: percent of total area comprised of green space, local human population density, and frequency of human traffic within 25 m of the feeder. Percent green space was calculated within a 250 m radius of each feeder using satellite images from Google Earth Pro (Google, Mountain View, USA). Images were analyzed in ImageJ (NIH, Rockville, USA) to calculate the total area of green space (vegetation, unpaved ground, and small areas of water) versus built space (buildings and paved areas such as sidewalks and parking lots). We calculated local human population density using population totals from the census block groups that feeders were located within (Data Access and Dissemination Systems, 2010), divided by the areas of these block groups (determined using 2010 US census maps and Image J; Data Access and Dissemination Systems, 2010). To quantify human traffic frequency, an observer recorded the number of people and motor vehicles passing within 25 m of the feeder during a 30‐min period. People or vehicles that loitered within 25 m of the feeder were counted once per minute to incorporate their continued presence into the data set. Observations were repeated at each site three times between 17 November 2017 and 14 December 2017, and averaged. All observations were performed within 4 hr of dawn on weekdays to ensure that our human activity estimates would be comparable to bird activity levels estimated through traditional observation methods, described below.

**Table 1:** Quantitative measures of human‐related disturbance at our three feeder deployment sites

Site	% green space	Human population density (people/km^2^)	Average motor vehicles/hr	Average pedestrians/hr
Campus	28.3	11,589.50	0	116.6
Residential	72.72	887.4	4	2
Nature conservancy	97.4	20.8	0	0

Note

Green space measures are a percentage of total area within a 250 m radius of each feeder. Human population densities are for the US census block group that the feeder was located within. Average vehicles and pedestrians per hour are for a 25 m radius area around each feeder.

We deployed feeders and collected pilot data from 29 August 2017 to 15 January 2017. This 140‐day period of feeder deployment allowed for both seasonality and weather to be assessed as dynamic factors in the study. The Campus feeder was deployed for 82% of this time period, the Residential feeder was deployed for 64% of the time, and the Conservancy feeder was deployed 71% of the time. We will use ANOVA, *t* test, and equivalence statistical analyses to interpret our data.

### 2.4 Traditional field observations

To compare the feeders’ data collection abilities to data collected via traditional methods using human observers, we performed four observations between 1 February 2018 and 23 February 2018 on each of the two feeders that received consistent bird activity earlier in the pilot period (Campus and Residential). Each observation consisted of a single observer watching the feeder with binoculars for a period of 30 min. Observers sat in a stationary position 20 m from the feeder or employed a nearby building as a blind in cases of extreme cold or when the front of the feeder was not visible from 20 m due to intervening vegetation or structures. At the beginning and end of each observation, the observer held up a card in front of the feeder's camera and triggered the proximity sensor to mark the set of data we would later compare their personal observations to. Observers recorded and identified by species (and sex, when possible) for each individual to visit the feeder, as well as any instances of incoming birds displacing the previous occupant at the feeder. Visits were recorded in order of occurrence for optimal comparison with feeder recordings of the same time period. This resulted in data on the number of visits to the feeder, the number of species to use the feeder, the relative numbers of visits per species, and displacement behaviors among and between species.

## 3 RESULTS

### 3.1 Overall feeder performance

Our three feeders were actively recording data, on average, for 72% of the total deployment period (campus feeder = 82%; residential feeder = 64%; nature conservancy feeder = 71%). In this time, they recorded 19,660 total image sets triggered by visits to the feeders. When triggered, feeder cameras took sets of 10 photos, with an average of 0.64 s between individual photos. The time from proximity sensor activation to camera activation was 2.8 s on average; this time to initial photo has been shorted to 1.7 s by changing the order in which the sensor activates the food‐dispensing servo and camera to prioritize the camera.

During the deployment period, feeders were occasionally removed for maintenance and repair or to avoid damage during extremely adverse weather conditions such as windstorms. Some data were also lost due to loss of battery power. Early in the deployment period, these gaps in data collection occurred more frequently, however, adjustments made to the physical design, power supply, sensors, and software during the deployment period have substantially improved feeder reliability.

### 3.2 Comparison of feeders to human observations

Feeders recorded an average of 7.4 (*SE* = 8.5) visits/30 min. observation by an average of 1.6 (*SE* = 0.9) species. Human observers recorded an average of 11.6 (*SE* = 10.5) visits/30 min. observation by an average of 2.4 (*SE* = 1.5) species. T tests comparing (a) number of visits observed by humans versus feeders and (b) number of species observed by humans versus feeders did not find significant differences between human and feeder performance (*F*‐ratio = 0.7879, *p* = 0.3897 and *F*‐ratio = 1.4483, *p* = 0.2488, respectively). We also performed two‐sample equivalence tests to determine the degree to which human and feeder data matched. For the test comparing the number of visits observed by humans versus feeders, we used an equivalence bound of 22.5 (25% of the mean of the human observations). This threshold was chosen because we expected some variation in these overall numbers due to the human ability to discriminate between repeat bird visits and a single long feeding bout, which the feeders currently lack due to their use of a simple proximity sensor trigger. Mean visits were found to be similar at this threshold (lower bound *p* = 0.0011, upper bound *p* < 0.0001, *SE* = 4.787). The equivalence test comparing number of species observed by humans versus feeders used an equivalence threshold of one species, which was chosen because it was the lowest possible detectable difference and because both feeders and human observers were known to occasionally miss a species. The results showed equivalence only for the upper bound, indicating that the current iteration of our feeders lacks a high degree of similarity to humans in the number of species they detect. (lower bound *p* = 0.347, upper bound *p* = 0.0070, *SE* = 0.623).

Species that human observers recorded but that were missed by the feeders during certain observations were black‐capped chickadees, tufted titmice, song sparrows, and house sparrows. Feeders may have missed feeding events due to technological issues such as the slight lag time between sensor activation and camera activation or the proximity sensor not activating appropriately when a bird arrived. Human observers also missed birds visiting the feeder; during one observation, a human observer missed visits by northern cardinal(s) that were recorded by the feeder. During another observation, both the human and feeder missed a visit by a house sparrow that was recorded on a separate video camera set up for comparison purposes.

### 3.3 Site differences

Our three deployment sites were selected for their differences in degree of human disturbance, both in terms of development and day‐to‐day activity. Campus was our most disturbed site, with the lowest percentage of green space, highest human population density, and significantly more pedestrians per hour than the other sites (Oneway ANOVA: *F*‐ratio = 33.98, *p* = 0.0005). The nature conservancy, as expected, was our least disturbed site for all three of these metrics. The residential site fell in the middle, although it had more cars passing near the feeder than either of the other sites (*F*‐ratio = 12.00, *p* = 0.008) due to the position of the feeder within 25 m of a road (Table 1).

Both total and average daily bird activity varied greatly depending on feeder location (campus feeder = 7,058 total, 61.4 per day; residential feeder = 11,569 total, 134.5 per day; nature conservancy feeder = 0 total, 0 per day), with the residential feeder receiving the most activity and the nature conservancy feeder remaining unvisited during the entire deployment period. Species composition of the feeding groups also varied between sites. Northern cardinals (*Cardinalis cardinalis*), black‐capped chickadees (*Poecile atricapillus*), eastern tufted titmice (*Baeolophus bicolor*), blue jays (*Cyanocitta cristata*), American goldfinches (*Spinus tristis*), and house finches (*Haemorhous mexicanus*) were present at both the campus and residential feeders to varying degrees. The campus feeder was also visited by downy woodpeckers (*Picoides pubescens*). The residential feeder was frequented by song sparrows (*Melospiza melodia)*, house sparrows (*Passer domesticus*), and Carolina wrens (*Thryothorus ludovicianus*) and occasionally visited by mourning doves (*Zenaida macroura)* and eastern towhees (*Pipilo erythrophthalmus)*, none of which were seen at the campus feeder.

Temporal feeding patterns also differed between sites. Specifically, a multiple regression model with species, site, and the interaction between species and site as explanatory variables, with hour of first feed for the day as the response variable found that birds typically fed earlier on campus than at the residential site (means equal 8.42 and 8.52, respectively; *F*‐ratio = 14.2956, *p* = 0.0002). This model included house finches, northern cardinals, tufted titmice, and black‐capped chickadees, the four species most commonly observed at both locations. The interaction term of this model was also significant (*F*‐ratio = 4.7735, *p* = 0.0028), indicating that inter‐site differences were larger for some species than others. Most notably, house finches were the latest to start feeding on campus, but the earliest feeders at the residential site.

**Figure 2:**
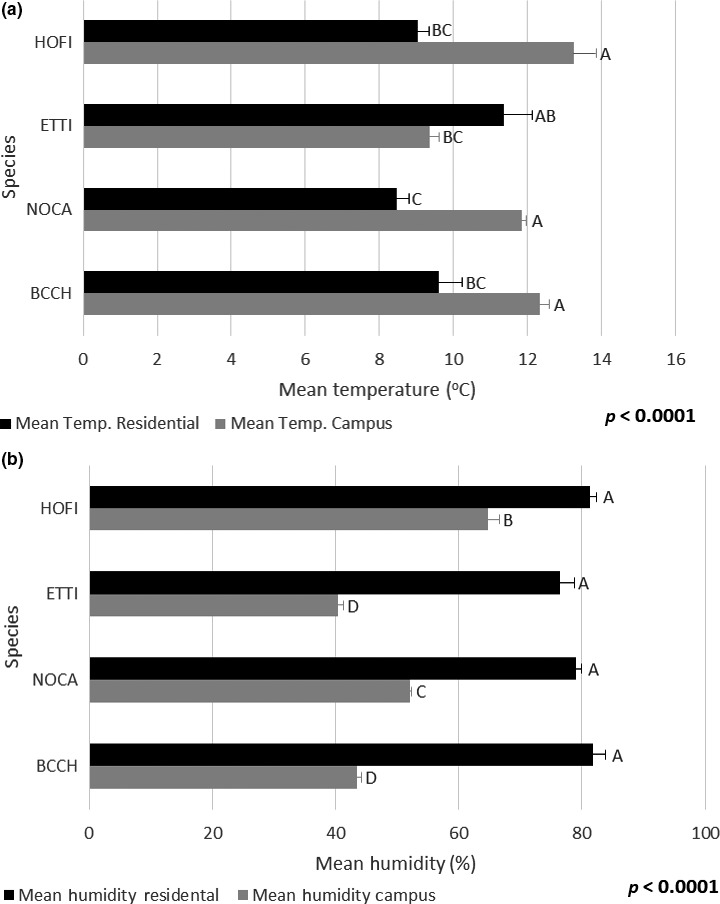
(A) Mean temperature (°C) of feeding per species by site. Post‐hoc tukey HSD test results run for both sites together. Significance between species shown. Species codes: HOFI =House finch (*Haemorhous mexicanus*), ETTI =Eastern tufted titmice (*Baeolophus bicolor*), NOCA =Northern cardinal (*Cardinalis cardinalis*), BCCH =Black‐capped chickadee (*Poecile atricapillus*). (b) Mean humidity (%) of feeding per species by site. Post‐hoc tukey HSD test results run for both sites together. Significance between species shown. Species codes: HOFI =House finch (*Haemorhous mexicanus*), ETTI =Eastern tufted titmice (*Baeolophus bicolor*), NOCA =Northern cardinal (*Cardinalis cardinalis*), BCCH =Black‐capped chickadee (*Poecile atricapillus*)

We used multiple regressions to analyze feeding patterns relative to temperature and humidity data from the four common species found at both sites, as listed above. The model with temperature as the response variable and species, site, and the interaction of species and site as the explanatory variables found that average feeding temperature at the campus site was significantly higher than at the residential site (averages equal 11.71 and 9.62 degrees Celsius, respectively; *F*‐ratio = 39.03, *p* < 0.0001). Some species’ average feeding temperatures also differed by site (*F*‐ratio = 13.66, *p* < 0.0001). For example, a post‐hoc Tukey HSD test found that, though tufted titmice fed at the lowest temperature of any of the four species at the campus location (9.38°C), their average feeding temperature at the residential location (11.37°C) was higher than any of the other species (Figure 2a). Similarly, our model with humidity as the response variable and the same explanatory variables showed that humidity varies significantly by site (averages equal 50.16°C and 79.66°C, respectively; *F*‐ratio = 856.94, *p* < 0.0001) and that species typically fed at different humidities at different sites (*F*‐ratio = 21.13, *p* < 0.0001; Figure 2b).

### 3.4 Species differences

The smart feeders proved themselves capable of recording a wide range of data. Over their 4.5‐month deployment, the feeders recorded 18,627 feeding events across 12 species of birds, as well as eastern gray squirrels. Common species included northern cardinals, house sparrows, tufted titmice, black‐capped chickadees, and song sparrows, with blue jays, house finches, and Carolina wrens also appearing fairly often. Additional, relatively infrequent (<50 observations) feeder visitations were recorded from downy woodpeckers, American goldfinches, mourning doves, and eastern towhees.

There were noticeable differences in feeder use across species within our data set. Some species fed more frequently than others (Table 2). Similarly, some species fed earlier than others (*F*‐ratio = 10.7353, *p* < 0.0001; Table 2). Weather also had species‐specific effects on feeder use across sites (multiple regression models, described in Site Differences, above: temperature, *F*‐ratio = 2.8264, *p* = 0.0371; humidity, *F*‐ratio = 30.0796 *p* < 0.0001). Average feeding humidity varied significantly across all four species, with house finches feeding at the highest relative humidity (77.68%) followed by northern cardinals (55.54%) and black‐capped chickadees (47.25%) at similar intermediate humidities, then tufted titmice at the lowest humidity (43.93%; Tukey‐Kramer HSD; Figure 2a,b).

**Table 2:** Total visits recorded by the feeder for all species that visited and hour of earliest feeding averaged across all visits made by each species

Species	# of Visits	Earliest Feeding Time
American goldfinch	46	9:00
Black‐capped chickadee	1553	8:05
Blue jay	239	8:11
Carolina wren	258	10:25
Downy woodpecker	21	10:30
Eastern towhee	1	8:00
Eastern tufted titmouse	1,189	8:29
House finch	838	8:08
House sparrow	4,468	8:47
Mourning dove	41	9:30
Northern cardinal	4,929	7:58
Song sparrow	4,392	7:35

**Figure 3:**
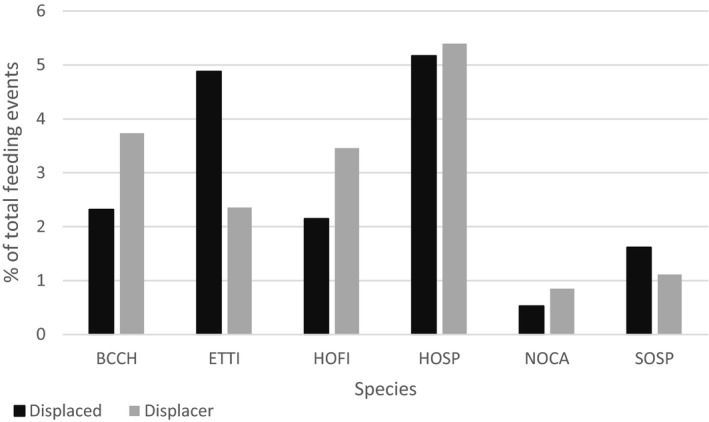
Percent of total feeds each species was displaced/displacer out of total feeding events. Species listed had>10 displacement events during study period. Species codes: BCCH =Black‐capped chickadee (*Poecile atricapillus*), ETTI =Eastern tufted titmice (*Baeolophus bicolor*), HOFI =House finch (*Haemorhous mexicanus*), HOSP =House sparrows (*Passer domesticus*), NOCA =Northern cardinal (*Cardinalis cardinalis*), SOSP =Song sparrows (*Melospiza melodia*)

Species also displaced each other at the feeding perch asymmetrically. Song sparrows and tufted titmice were more likely to be displaced than to displace other species (*p* = 0.0234 and *p* = 0.0007, respectively; binomial probability test), while black‐capped chickadees and northern cardinals were more likely to be the displacer (*p* = 0.0167 and 0.0249, respectively; binomial probability test; Figure 3). Species also differed significantly in how commonly they were involved in displacement events overall (χ^2^ = 26.1068, *p* = 0.0020; Pearson chi‐squared probability test). For example, house sparrows were much more likely to be part of a displacement event than song sparrows (11.5% of observations versus 2.8% of observations; Table 3), despite a similar number of observations of both species from the same feeder (house sparrows = 4,468 observations, song sparrows = 4,392 observations, residential feeder; Table 3).

**Table 3:** Count and percent of feeds where species was displaced, or was the displacer for both locations

Species	*n* of Total Feeds	*n* of Feeds Displaced	*n* of Feeds as Displacer	% of Feeds Displaced	% of Feeds as Displacer
BCCH	1553	36	58	2.32%	3.73%
ETTI	1,189	58	28	4.88%	2.35%
HOFI	838	18	29	2.15%	3.46%
HOSP	4,468	231	241	5.17%	5.39%
NOCA	4,929	26	42	0.53%	0.85%
SOSP	4,392	71	49	1.62%	1.12%
AMGO	46	2	1	4.35%	2.17%
BLJA	239	1	0	0.42%	0.00%
CAWR	258	7	2	2.71%	0.78%

Note

Only species with >10 displacement events during study period listed. Species codes: BCCH =Black‐capped chickadee (*Poecile atricapillus*), ETTI =Eastern tufted titmice (*Baeolophus bicolor*), HOFI =House finch (*Haemorhous mexicanus*), HOSP =House sparrows (*Passer domesticus*), NOCA =Northern cardinal (*Cardinalis cardinalis*), SOSP =Song sparrows (*Melospiza melodia*).

## 4 DISCUSSION

### 4.1 Smart feeder successes and limitations

The studies described here show that the PASSER smart feeders were able to successfully achieve the objective of large‐scale automated feeding data collection of songbird's environment–behavior interactions. The feeders collected reliable environmental and behavioral data across a variety of weather conditions with relatively minimal hands‐on maintenance, generating a large and varied data set mapping feeder activity with high resolution and accuracy. In the course of several months, the PASSER feeders recorded almost 20,000 feeding events and over 170,000 individual photographs, generated from 2 of the 3 active feeders.

Though the feeders were able to collect data on over 72% of the time period, they were not without issue. However, after iterations of the feeder correcting for their vulnerability to weather, lack of sufficient battery power, and other general design issues, the overall performance and reaction speed of feeders were improved (feeders collected data 89% of the time in the final month of the study). Despite these improvements, running a large array of these feeders would require some technical skill and the time to devote to feeder maintenance due to the need for fine‐tuning to specific circumstances and additional troubleshooting.

Feeders were highly successful in semi‐urban and residential locations; however, they experienced extremely low use in more rural locations. This suggests that both species and habitat type may be important factors when considering whether these feeders are appropriate for a given research study (Clergeau, Savard, Mennechez, & Falardeau, 1998). Due to their unusual design and the constraints of feeders in general, individual animals with limited experience using feeders may be reluctant to use them, potentially producing bias in a data set or restricting its overall generalizability (Cooper, Hochachka, & Dhondt, 2007; Melles, Glenn, & Martin, 2003; Robb, McDonald, Chamberlain, & Bearhop, 2008).

In addition, it is worth noting that the feeders produced an overwhelmingly large amount of data, with the photographs having to be logged and identified by trained observers, making the time to process these data sets substantial. That said, the fixed angle of the camera relative to feeding birds makes these ideal for machine learning and automated photographic coding (Kelling et al., 2013; Yoshihashi, Kawakami, Iida, & Naemura, 2017). We are currently developing neural network software to aid in identification of subjects based on natural markings. This software has already shown great potential to identify both species and sex of birds from single photographs.

Current designs of our feeders are unable to identify and target birds on an individual level, a capability that other computerized feeding units using RFID systems currently have (such as: Firth, Voelkl, Farine, & Sheldon, 2015; Voelkl, Firth, & Sheldon, 2016). This makes our current feeders unsuitable for studies needing to identify individual birds. However, we anticipate our neural network software will soon have the matrices required for individual identification.

### 4.2 Smart Feeders in comparison to traditional methods

Traditional first‐person observational techniques and smart feeders each have strengths and weaknesses, and as such may often work best as complementary, rather than alternative, data collection techniques. The average number of species recorded by human observers, though not statistically different from those recorded by the feeders, was also not statistically similar. While neither feeders nor human observers consistently recorded every visit to the feeder, human observers tended to record more species than the feeders did. Feeders tended to miss species that the human observers noted as making quick feeding visits. Such rapid visits were most likely shorter than the 1.7 s trigger speed of the feeder camera and may account for this discrepancy. Thus, the current version of the feeder may not be appropriate for studies that require accurate recordings of all species or are focused on species that tend to perform rapid feeding visits rather than remaining at the feeder to eat. Additionally, as discussed above, the feeders’ motion‐triggered camera feature does not allow us to discriminate reliably between multiple consecutive visits by birds of the same species and a continuous feeding bout by a single individual who triggers the camera repeatedly, unless individuals in the population have been previously marked. Such discrimination may not be necessary for all studies, as multiple records, whether of one bird or many, can provide an estimate of the relative use of feeders by different species in the area and under various conditions. Continued development of these feeders, including the addition of recognition software, will begin to resolve some of these issues.

Nonetheless, our smart feeders have some distinct advantages over human observers, not the least of which is their ability to collect data consistently over long periods of time, regardless of weather, location, and time of day. Because of this, smart feeders are able to assemble not only comprehensive observational data sets over long periods, but are also able to record relatively large numbers of otherwise infrequent or unusual occurrences, including such activities as displacements, co‐feeding behaviors, and uncommon or transitory species feeding events. Unlike feeder designs that rely on RFID tags, these data can be collected without previously capturing and marking all individuals in the target population. Thus, these feeders enable the continuous monitoring of large numbers of birds or on species where capture (to be marked with RFID or color bands) may not be practical.

Additionally, because feeders keep an up‐close photographic record of species that visit, mistaken species identification is not an issue in the same way that it can be with human observers, who may be unfamiliar with certain species, momentarily distracted, or unable to discriminate similar species while maintaining a non‐disruptive distance from the feeder. Because the feeders are almost entirely non‐disruptive; due to their passive nature and inanimacy, once birds have become accustomed to their presence they do not alter patterns of behavior in the way that a human observer may (Farmer et al., 2014; Gibson et al., 2015; Jiguet, 2009). As such, they are also able to interact, with a high degree of stimulus, with feeding birds. This makes it possible to present controlled stimuli directly to subject animals, giving rise to a number of potential experimental interventions and treatments that would otherwise have been extremely difficult to conduct in the wild (Camín, Martín‐Albarracín, Jefferies, & Marone, 2016; Greenburg, 1984).

### 4.3 Data collected from our smart feeders

Our smart feeders recorded a wide range of species feeding. They also captured different patterns of behavior across these species, including overall numbers of visitations, timing of visitations, and propensity for displacing or being displaced during feeding. Feeder data also showed that species’ individual feeding profiles differed in regard to basic weather conditions. Given the ongoing shifts in weather patterns and climate (Fontaine, Decker, Skagen, & Riper, 2009; Root, Price, Hall, & Schneider, 2003), mapping interactions between meteorological patterns and animal behavior are of ever‐increasing importance to both understand the underlying connections that influence the day to day lives of animals and as a means to predict vulnerabilities that may precede species die‐offs or environmental collapse.

Data collected by feeders showed strong and reliable differences across sites. As noted, three smart feeders were placed at three sites: Campus, Residential, and Nature conservancy. Sites were in relatively close proximity to one another (<3 km), and as such did not experience any substantial differences in climate or weather. However, they did exhibit large differences in the overall nature and degree of anthropogenic disturbance. Campus and residential sites had substantial overlap in the types of species that visited and the time pattern of these visitations, though the residential feeder recorded both more frequent feeding events and a wider range of species. In addition, the four species we analyzed (house finches, black‐capped chickadees, tufted titmice, and northern cardinals) all exhibited differences in earliest average daily feeding time both overall and relative to each other. Such differences may be related in part to local environmental variation. T tests comparing temperature and humidity of all recorded visits to the feeders irrespective of species showed that both measures differ significantly between sites (temperature: *t*‐ratio = 35.6504, *p* < 0.0001; humidity: *t*‐ratio = −75.1974, *p* < 0.0001), suggesting either that all species visiting the feeders shifted their feeding times across sites in the same direction or that microclimate differences exist between sites (Figure 2a,b). Differences in feeding behavior between sites may also be related to interspecific interactions; competition between species may impact which species feed at certain times.

Feeders excelled at collecting uncommon behavioral events. In the course of 140 days of analysis, the feeders recorded 450 displacement events, during which one bird would be displaced/replaced by another within the timeframe of three consecutive photos from a single triggering event (~1.5 s; Table 3). Analysis showed strong species‐related patterns with some species preferentially replacing others (Figure 3).

### 4.4 Future directions

Applying computer automation to traditional field‐based ethological studies focusing on the interaction between the environment and avian feeding behaviors has not previously been widely available due to limitations of technology, cost and accessibility/expertise. The work we have piloted with the PASSER project demonstrates that techniques to address environment–behavior questions are not only coming of age but also go beyond simply easing the process of data collection to enable novel research lines and methodologies that would not have otherwise been easily conducted.

The flexibility inherent in the “smart feeder” allows them to be easily modified or customized. Perches and placement can be altered to provide access to a variety of species, including species that feed on the ground or on the wing. Control of food delivery enables precise dietary modulation, including supplementation, restriction, and the delivery of different food types or chemically modified foods to different species or even individuals, through incorporation of RFID or similar devices. Interactive presentation of stimuli can be made across multiple sensory modalities through incorporation of audio or video displays. Even olfactory cues could be dynamically modified with relative ease, if needed.

It is important to note that embedded “smart technology” in ethological studies need not be limited to feeders. Our laboratory is already at work on “smart nests” that incorporate many of the same design principles discussed above, including solar power and interactive recordings. These “smart nests” also incorporate active sampling devices, allowing as needed capture and containment, and collection of microbial, fecal, and/or feather samples. Zárybnická, Kubizňák, Šindelář, and Hlaváč (2016) has already constructed similar nest boxes to study cavity‐dwelling animals.

Overall, these smart feeders have allowed our research team to more closely and accurately investigate feeding behaviors in wild songbirds, comparing them across both spatial and temporal ranges. They have enabled recording and analysis of both unpredictable and uncommon events, and in so doing have provided us with new insights into environment–behavior interactions. We anticipate further use of these and similar models in ours, and many other's future studies, incorporated as a supplement to traditional ethological observational methods.

## CONFLICTS OF INTEREST

Authors declare no conflicts of interests.

## AUTHORS CONTRIBUTION

JD and SF conceived the idea behind the project. CP, AR, and JD designed methodology; CP, SF, and JD collected the data; CP, SF, and JD analyzed the data; CP, SF, and JD wrote the manuscript. All authors contributed critically to the drafts and gave final approval for publication.

## DATA ACCESSIBILITY

Data related to this manuscript will be made available on Mendeley Data.

## Figures and Tables

**Figure A1:**
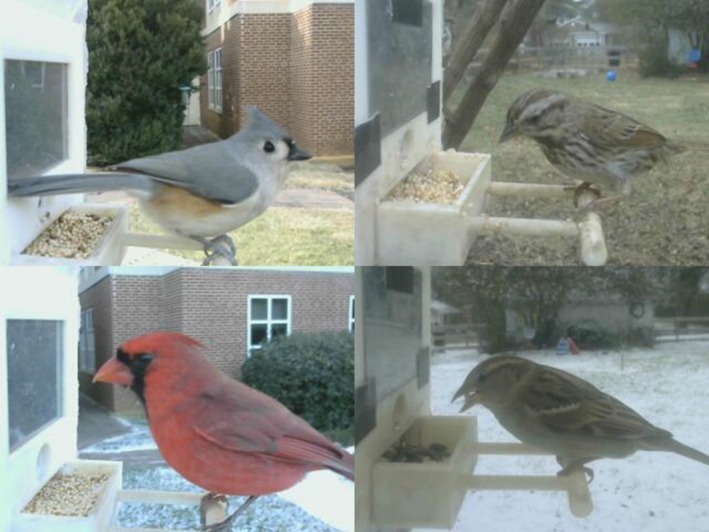
View from our PASSER smart feeder’s camera. Top left: Eastern tufted titmice (*Baeolophus bicolor*) at the campus feeder. Bottom left: Male northern cardinal (*Cardinalis cardinalis*) at the campus feeder. Top right: Song sparrow (*Melospiza melodia*) at the Residential Feeder. Bottom Right: Female house sparrow (*Passer domesticus*) at the residential feeder

**Figure A2:**
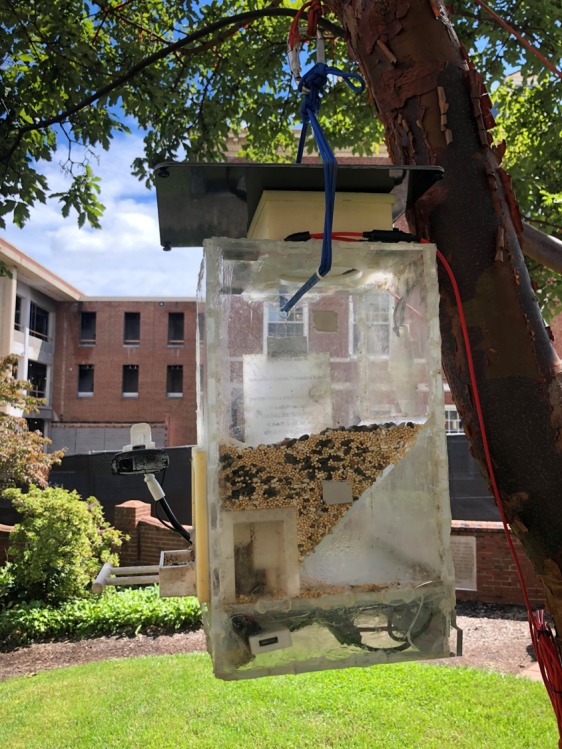
A side view of our PASSER smart feeder at the campus location

## APPENDIX 1

List of materials and equipment used to design and assemble our feeder units

Feeder dimensions: 20.2 × 20.2 × 35.6cm

Feeders structure: 0.635 cm thick acrylic sheets, Stratasys Mojo QuickPack ABS Print Engine (Stratasys, Eden Prairie, USA), and MakerBot PLA Filament (MakerBot, New York, USA)

Designed using: AutoCAD (Autodesk Inc.) and TinkerCAD (Autodesk Inc.)

Machines used: ShopBot Computer Numerical Control spindle bit machine (“CNC”; ShopBot, Durham, USA), Stratus Mojo (Stratasys, Eden Prairie, USA), and MakerBot Replicator 2 (MakerBot, New York, USA)

Power source: Voltaic Systems V44 Lithium Ion Batteries and Voltaic Systems 9‐watt Voltaic Solar Cells (Voltaic Systems, New York, USA)

Computer: Raspberry Pi Zero ‐ Version 1.3 with Raspbian operation system (8 GB SD Card w/ Stretch Lite; Product ID: 2820; Raspberry Pi, Cambridge, UK; Adafruit Industries, New York, USA)

Computer accessories and sensors: PiTFT Plus 480 × 320 3.5" TFT+Touch screen for Raspberry Pi (Product ID: 2441), DHT11 basic temperature‐humidity sensor + extras (Product ID: 386), Adafruit DS1307 Real Time Clock Assembled Breakout Board (Product ID: 3296), VCNL4010 Proximity/Light sensor (Product ID: 466), Micro servo (Product ID: 169), USB cable ‐ USB A to Micro‐B (Product ID: 592), Breadboarding wire bundle (Product ID: 153), Break‐away 0.1" 2 × 20‐pin Strip Dual Male Header (Product ID: 2822), Adafruit Perma‐Proto Quarter‐sized Breadboard PCB (Product ID: 1608). All from Adafruit Industries (New York, USA).

Data Storage: SanDisk Ultra Fit USB 3.0 Flash Drive (32 gb; Western Digital Technologies, Milpitas, USA)

Camera: Logitech C270 HD Webcam (Logitech, Romanel‐sur‐Morges, Switzerland)

Additional Materials (general): Acrylic glue, silicone, hydrophobic spray, electrical tape and Gorilla tape
